# Revisiting the timetable of tuberculosis

**DOI:** 10.1136/bmj.k2738

**Published:** 2018-08-23

**Authors:** Marcel A Behr, Paul H Edelstein, Lalita Ramakrishnan

**Affiliations:** 1 McGill International TB Centre, Infectious Diseases and Immunity in Global Health Program, McGill University Health Centre Research Institute, Montreal H4A 3J1, Canada; 2 Department of Pathology and Laboratory Medicine, Perelman School of Medicine, University of Pennsylvania, Philadelphia, PA 19104, USA; 3 Molecular Immunity Unit, Department of Medicine, University of Cambridge, MRC Laboratory of Molecular Biology, Cambridge CB2 0QH, UK

## Abstract

Tuberculosis has a much shorter incubation period than is widely thought, say **Marcel A Behr and colleagues**, and this has implications for prioritising research and public health strategies

Between a quarter and a third of the world’s population are estimated to be latently infected with *Mycobacterium tuberculosis*.^1^ The 2018 World Health Organization resource page for tuberculosis (TB) states: “On average, 5-10% of those who are infected will develop active TB disease over their lifetime.” Other authorities use terms such as “dormant” or “alive but inactive” (supplementary box 1).

Because “reactivated” TB is contagious, eradicating latent infection is a cornerstone of global TB control^2^ and achieving a better understanding of latent infection is deemed a research priority.^3^ ^4^ The word latent has both biological and medical definitions. The biological concept of latency is that of a resting stage, hidden until circumstances are suitable for development. The medical definition is simply a pathological process in which symptoms are not yet manifest. The TB clinical community has long used the apposition of latent TB infection and reactivation, clearly applying the biological definition.

The importance attached to latency is reflected in a major push from research funding agencies to understand the biology and epidemiology of latent TB infection and to develop drugs that specifically treat latent infection, aiming for global TB eradication (supplementary box 2). Multiple longitudinal epidemiological studies, however, show that the majority of TB disease manifests soon after infection, with disease rarely occurring more than two years after infection. (We use the term “remote infection” to describe infection preceding active TB by more than two years.) The vast burden of global TB is, therefore, from recently transmitted infection. Only in countries with a low TB burden, where ongoing transmission is minimal, is TB from remote infection a substantial contributor to the active TB burden.^5^ Importantly, most such TB cases do not generally result in major disease outbreaks,^6^ ^7^ probably as a result of well functioning public health systems.

Appreciating the natural history of infection and disease should help us to strategise for the global eradication of TB and to design vaccine efficacy trials. Furthermore, the natural history of TB does not support the many terms currently used to describe the various phases of TB infection. These terms are not only confusing, but even misleading. We suggest using just three simple terms—tuberculous reactivity, primary infection, and active TB (box 1).

Box 1 **Suggested simplified terms**

*Tuberculous reactivity*—Indirect evidence of present or past infection with *Mycobacterium tuberculosis* as inferred by a detectable adaptive immune response to *M tuberculosis* antigens (on tuberculin skin test or interferon gamma release assay) in an asymptomatic person

*Primary infection*—Evidence of new tuberculous infection, obtained with a tuberculin skin test conversion or a new positive interferon gamma release assay, which may be asymptomatic or accompanied by transient fever, erythema nodosum, elevated erythrocyte sedimentation rate or characteristic roentgenographic abnormalities

*Active tuberculosis—*Evidence of progressive disease of the lung and/or other organs generally accompanied by a positive culture for *M tuberculosis* and/or roentgenographic findings and/or histopathology consistent with TB

## TB incubation studies from the pre-antibiotic era

**Fig 1:**
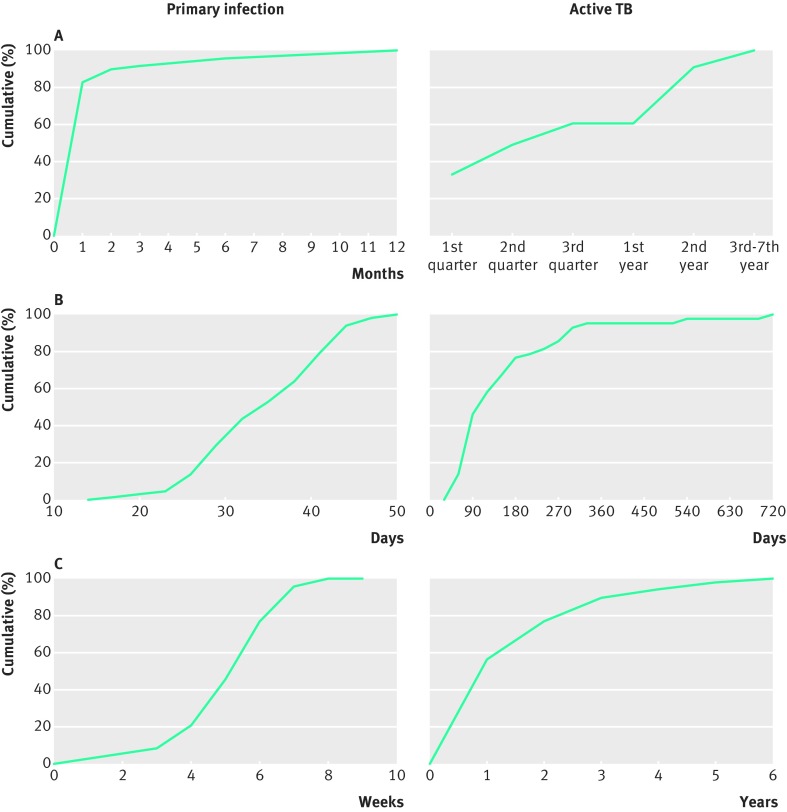
Incubation periods and cumulative percentage of patients developing primary infection or active TB in three different studies. A) Incubation periods are measured from the time of tuberculin skin test conversion to the first clinical indication of primary infection (n=130) and active TB (n=33), respectively. Data from Gedde-Dahl.^8^ B) Incubation periods are measured from the time of exposure to the first clinical indication of primary infection and active pulmonary TB, respectively (n=64 for each). Data from Poulsen.^9^ C) Incubation period of primary infection is measured from the time of tuberculin skin test conversion to the first clinical indication of primary infection (n=48). Incubation period of active TB is measured from the time of onset of primary infection to the development of active TB (n=106). Data from Wallgren.^10^

Three longitudinal studies of TB acquisition and progression were conducted before the widespread use of antibiotics in Norway and Sweden.^8^ ^9^ ^10^ Careful monitoring by astute clinicians allowed for a reproducible timeline from acquiring the primary infection to developing active TB. Poulsen, while working at the TB station in the Faroe Islands from 1939 to 1947, was able to pinpoint the time of exposure to TB to a two week period and often to a single day.^9^ Thus, he determined the incubation period of primary infection—new tuberculous reactivity often accompanied by characteristic clinical features (box 1)—to be under six weeks (fig 1A, left). In this same cohort, the incubation period of active TB was typically 3-9 months and almost always under two years (fig 1A, right). Wallgren, working in Stockholm, similarly found that active pulmonary TB generally developed within 1-2 years of exposure (fig 1B, right).^10^ Finally, Gedde-Dahl, who monitored people regularly for tuberculin skin test (TST) conversion (that is, the point at which results of the test switch from negative to positive) and then for development of active disease, found a similar incubation period for the development of active TB, usually 3-9 months and rarely beyond two years of newly documented tuberculous reactivity (fig 1C, right).^8^

## TB incubation studies from the post-antibiotic era

**Fig 2:**
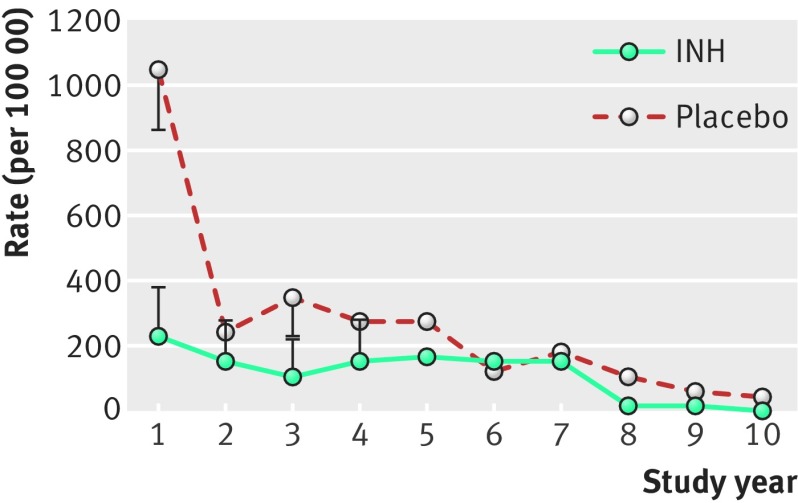
Rates and incubation period of active TB in tuberculin positive (induration ≥5 mm) household contacts of patients with recently diagnosed TB. Half of the contacts were treated with isoniazid and half with placebo for one year, then observed for another 10 years. The total number of people for both groups was 13 176. Error bars show 95% confidence intervals. Data from Ferebee.^11^

When isoniazid became available in 1952, clinicians were interested in using it to treat TB disease and as a chemoprophylaxis agent to prevent the development of TB disease after infection was diagnosed. In 1970, Ferebee published a review of the controlled isoniazid chemoprophylaxis trials conducted in the United States between 1956 and 1966 (fig 2).^11^ Examination of the placebo recipients shows that, as in the older studies, the likelihood of developing TB disease after infection dropped precipitously after the first year, leaving a tail of what might be considered “reactivation” TB. Examining those who received isoniazid provides additional insights into this tail. Isoniazid was given for 12 months after infection, and its efficacy in preventing TB disease is reflected in a fivefold reduction at year one. After year one, the rates of TB disease were no different between placebo and isoniazid arms, indicating that newly acquired infections, rather than reactivation of the original infection, were substantial contributors to this tail. Similar findings were reported in the recent isoniazid prevention trial in South African goldminers, where a transient decrease in cases during the intervention period was followed by a convergence of the study groups.^12^ Thus, the true incidence of TB occurring remotely after infection (more than two years) may be lower than was surmised in the pre-antibiotic era.

A re-examination of isoniazid trial data also challenges the assumption that people with roentgenographic evidence of fibrotic lung lesions of likely tuberculous origin are at higher risk of “reactivation TB.” In a 28 000 person trial of preventive therapy, which documented a clear medical benefit for isoniazid, the risk of TB disease in placebo recipients did not remain high over time; rather it was highest in the first year after enrolment and declined sharply thereafter,^13^ following the same timeline as the general cohort of patients with new positive TST results.^8^ ^9^ ^10^ ^13^

Several other studies support the inference that TB occurring remotely after primary infection can be due to newly acquired infection rather than reactivation. Studies using guinea pig inoculation (an exquisitely sensitive assay in which even one viable TB bacillus causes lethal disease) found that, in the pre-antibiotic era, 96%-98% of visible calcified tuberculous lung lesions in people who died of causes other than TB were sterile.^14^ ^15^ This finding is consistent with those of the epidemiological studies and is reinforced by two subsequent studies (also in the pre-antibiotic era) that performed detailed histopathological analyses of the lungs of people who died from TB to assess whether the cause was reactivation of their old foci or newly acquired infection.^16^ ^17^ Terplan found that 90% of 51 patients aged over 40 died of new exogenous infection without involvement of the previous focus of infection.^17^ Similarly, Canetti and colleagues ruled out endogenous reactivation in 67% of 69 cases; in the remaining 33% they could not definitively rule it out because a small calcified primary focus could easily be missed in a lung infected with TB.^16^ Canetti et al also took advantage of the fact that antibiotics had been introduced, and resistance had developed, by the time they submitted the paper in 1971. They reasoned that if reactivation of remote TB was responsible for most active TB infections, then older people should have a much lower frequency of drug resistant TB than younger patients. To test this, they examined the proportion of isolates with drug resistance in sequential age cohorts, ranging from 15 to ≥60 years. Although they found that resistance decreased with age, the difference was minimal; 9.2% of all 9456 patient isolates were resistant to isoniazid, streptomycin, or aminosalicylic acid, compared with 7.6% of the 1996 isolates from those aged 60 and older, which supports reinfection rather than reactivation in most of these patients.

In sum, both histopathological and epidemiological approaches indicate a far greater role for exogenous infection than reactivation of primary TB.

## Has the incubation period of TB changed?

Could the natural history of TB have changed since the earlier studies were performed so that the median incubation period is now longer? Three studies (two from the Netherlands and one from Canada) show that the incubation period of TB remains unchanged in the 21st century.^18^ ^19^ ^20^ Sloot and colleagues identified patients with recent active TB in Amsterdam and monitored their TST positive contacts who did not take isoniazid prophylaxis for 10 years (2002-11).^20^ They found that 75% of active TB cases in contacts occurred within one year of diagnosis of TB in the index case and 97% within two years (supplementary figure 1). The study confirmed that children and adolescents were at greater risk of developing active TB, but the timeline of developing TB was the same in all age groups—predominantly in the first year (supplementary figure 1).

The other two studies combined molecular fingerprinting with epidemiological methods to assess the incubation period more accurately. Borgdorff and colleagues identified secondary TB cases among contacts of index patients and confirmed strain identity between the two using molecular fingerprinting methods.^18^ They found that the median incubation period was 1.3 years (95% confidence interval 1.1 to 1.4 years, range 0-12.8 years). The probabilities of developing disease within one, two, and five years were 45%, 62%, and 83%, respectively. A Canadian study tracked transmission of infection during an outbreak using a time labelled genome phylogeny of the *M tuberculosis* strains to estimate the time of infection for each of the secondary cases.^19^ The majority of the 50 secondary cases resulting from this outbreak presented with TB disease within two years of infection (supplementary figure 2).

Because both studies used molecular methods to track transmission and were conducted in an otherwise low incidence setting, these results unambiguously confirm the previously described timeline. In summary, the typical incubation period of TB disease has not changed and remains a few months to two years. The importance of recent infection as a risk factor for active TB was emphasised recently by Houben and Dodd in a modelling paper that provided both overall estimates of latent TB infection and the subset infected within two years.^1^

## Is there a late spike of TB disease?

**Fig 3:**
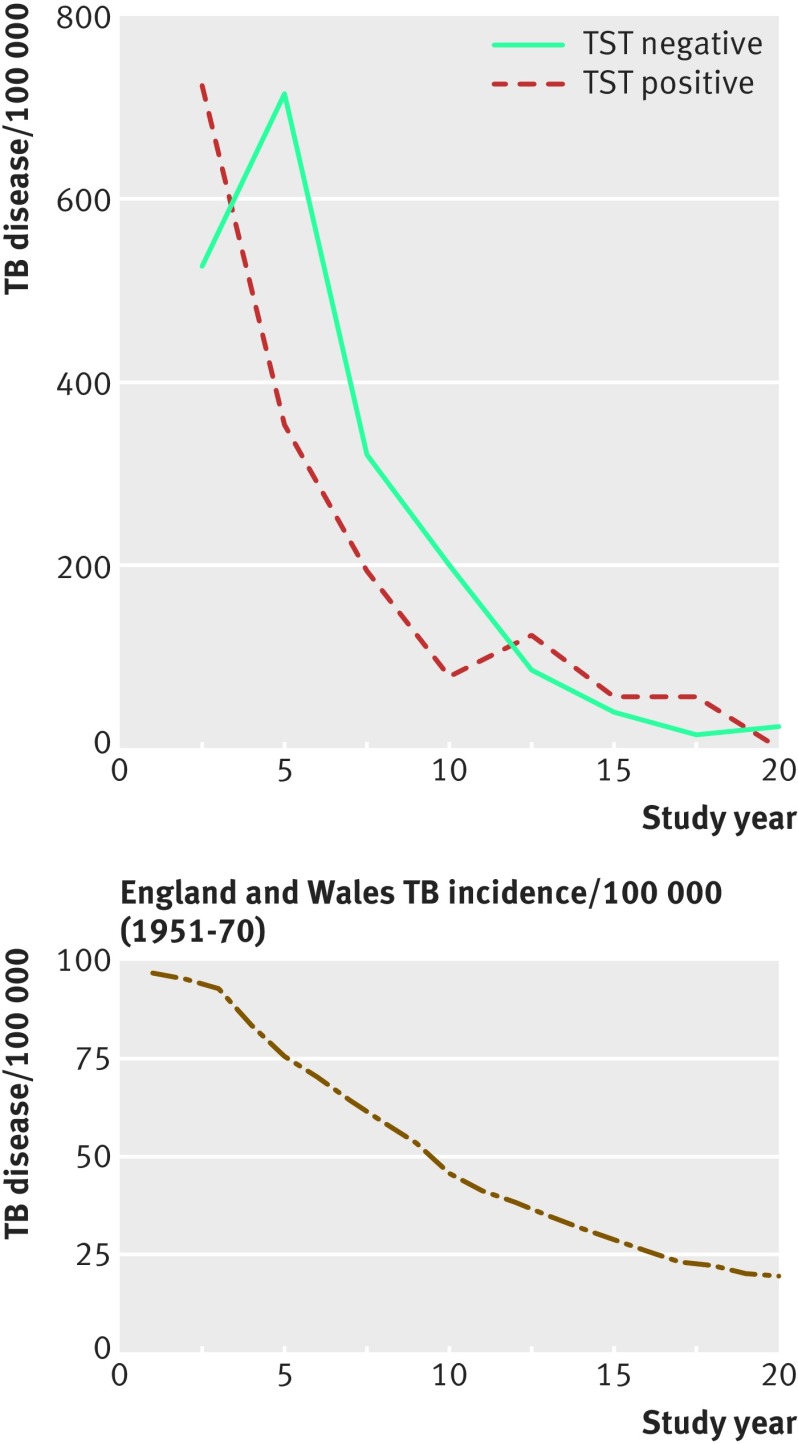
The incidence of active TB in 8838 British adolescents who were TST positive (≥15 mm induration) or TST negative, observed over 20 years, from 1951 to 1970 (top panel). The bottom panel shows the TB disease incidence rates for the same period in England and Wales. Data from Hart and Sutherland.^21^

Reactivation TB is thought to occur most frequently later in life when immunity wanes or intercurrent illness occurs. If this were the case, we would expect a rise in TB incidence decades after infection, and this would have been missed by the aforementioned studies that monitored people up to 10 years at most. The antibiotic resistance data from the Canetti study go against a late spike in disease.^16^ To examine this more rigorously, we looked at longer term epidemiological studies. A 20 year study followed TST positive and TST negative adolescents in England and Wales assigned to the control arm of a BCG vaccine trial (fig 3, top panel).^21^ This study was carried out from 1951 to 1970, a period of a sharp decline in TB incidence (fig 3, bottom panel). Importantly, the study showed that the fall was the same for both TST positive and TST negative people, with no late spike in disease (fig 3, top panel).

**Fig 4:**
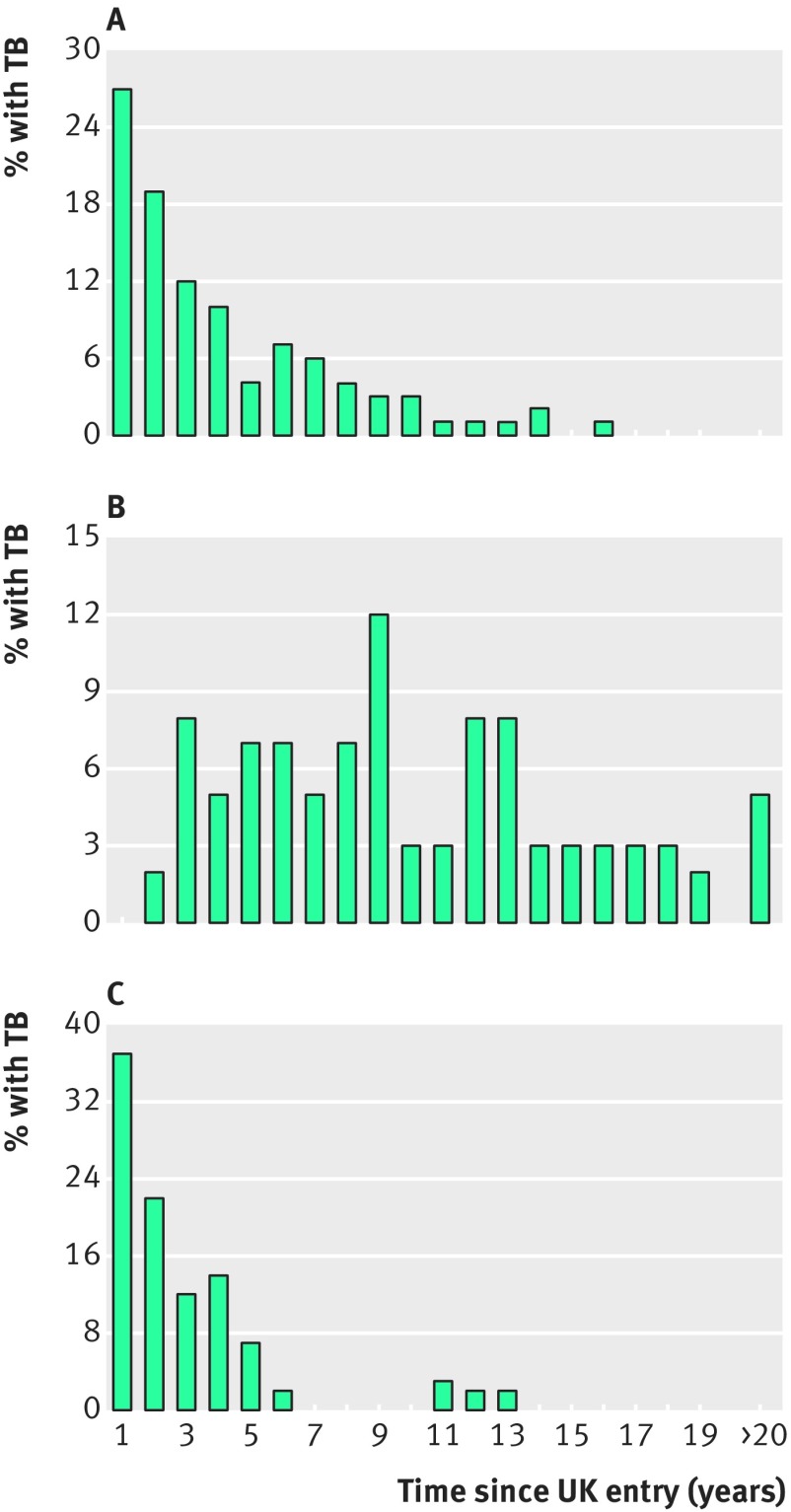
Time of onset of active TB in Asian immigrants to the UK, some of whom went to their home country and back to the UK before their onset of TB. A) Time of onset of TB in 128 immigrants who neither left the UK nor had known contacts with TB in the UK before receiving a diagnosis of TB. B) Time of onset of TB in 59 immigrants who visited their home countries and had no known contacts with TB in the UK before their diagnosis of TB. Time to onset is based on the time of initial UK entry. C) Same group shown (B), but with the time to onset measured by the time from re-entry into the UK after their Asian visits. Data from McCarthy.^22^

In another revealing study, McCarthy followed people who had migrated from Asia to London for more than 20 years, stratified by whether they had remained in the United Kingdom or had returned to Asia to visit friends or relatives (fig 4).^22^ In those who never returned to Asia, the majority of TB cases occurred in the first two years after arrival, with a steady steep decline thereafter and no late peaks (fig 4A). By contrast, those who visited their country of origin after initial arrival in the UK had an apparently steady rate of TB disease over the study period (fig 4B). When the time of re-entry after the re-visit was considered, however, disease occurred predominantly early (fig 4C).

**Fig 5:**
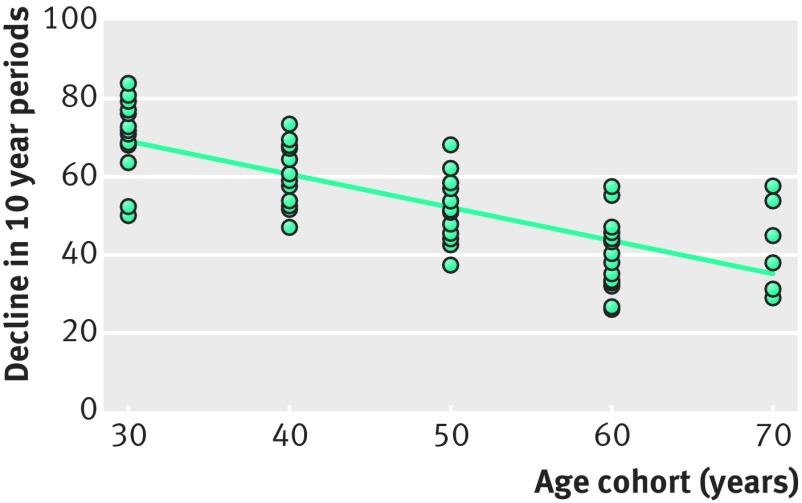
Decreasing rates of TB in different age cohorts of men, based on the TB incidence rate declines for Norwegian men over a 10 year period, for the years 1946 to 1974. The incidence of TB decreased by around 60% in the same group of 40 year old men, from one decade to the next. During this period the annual rates of TB disease in young men were 2.5 to 6/100 000. Redrawn from original figure in Wiker et al.^23^

Finally, Wiker and colleagues specifically tested the hypothesis that TB incidence increases with age by analysing the incidence of TB over 20-30 years in Norwegian men stratified into 10 year birth cohorts, from 1879-88 to 1959-68.^23^ Surprisingly, they saw a decreased incidence over the 10 year observation periods in all age cohorts (fig 5).^8^ ^9^ ^10^ ^20^ In sum, these studies show that, contrary to the prevailing view, TB does not have a bimodal distribution separating primary progressive disease from reactivation disease. Rather, the low rate of TB disease many years after infection continues to dwindle with time.

## What does the presence of TB immunoreactivity really mean?

In light of the findings that the risk of TB drops precipitously after the first couple of years and continues to drop further, we revisited the assertion that a quarter of the world’s population is latently infected with *M tuberculosis*. This statistic is derived from the finding that about a quarter of the world’s population exhibits immunoreactivity to TB, as shown by a positive TST or interferon gamma release assay (box 1).

The basis of adaptive immunity is that a memory response does not require the inciting pathogen to remain present. Therefore, TB reactivity must encompass both current and past infections. But is there evidence that TB reactivity persists after *M tuberculosis* is cleared? Several papers suggest so. Atuk and Hunt examined persistence of TST positivity at the end of one year of isoniazid treatment of asymptomatic, TST positive hospital employees.^24^ Among recent converters (less than one year), only five of 20 people remained TST positive to the same extent; the rest became TST negative or positive to a smaller extent. By contrast, all 17 people who had been asymptomatically TST positive for more than a year remained so after the year of isoniazid treatment. A study in naval officers had virtually identical findings.^25^ Almost all people who had been TST positive for only a few weeks at the start of isoniazid treatment reverted within three months; all who had been positive for more than one year remained positive at the end of the year’s treatment. These findings are consistent with TB immunoreactivity being retained well after infection is cleared. The more stable immunoreactivity of people with long term TST positivity is consistent with immunological memory being more robust and long lasting when the infection lingered longer before being cleared. This conclusion is corroborated by a study that looked for TST reversion after treatment of active TB, in which TST positivity was retained in all 38 patients even though they had completed a treatment regimen associated with <3% recurrence at five years.^26^

Together, these studies indicate that the reported burden of latent TB infection is overestimated, as it reflects immunoreactivity to either past or present infection. Not only are most TST positive people not at higher risk of TB, but multiple studies have shown that they may be protected against TB disease on subsequent exposure to infection (supplementary figure 3).^27^ ^28^ ^29^

## Summary and implications

Asymptomatic *M tuberculosis* infection can result in disease decades later, the most dramatic known example being a case of father to son transmission in Denmark with a 33 year interval between infection and disease, which was confirmed on genome sequencing.^30^ But the longitudinal studies that we have examined support a median incubation period of a few months to two years, with only a small proportion of people getting disease later. There is no evidence or epidemiological basis for a bimodal curve differentiating primary progressive TB from reactivation TB.

Furthermore, the epidemiological data do not support the existence of a special bacterial state (such as dormancy) during the asymptomatic phase of TB, no matter how prolonged. Whole genome sequencing of the bacteria that the Danish patient harboured for 33 years,^30^ and other isolates that were cultured after decades long incubation periods, found the same mutation frequency (0.2-0.3 single nucleotide polymorphisms per genome per year) as seen in outbreak strains.^31^

We must, therefore, recognise that the respective contribution of recent and remote infection differs in high and low transmission settings.^5^ In high endemicity areas, the vast majority of disease burden is accounted for by newly acquired infection.^1^ In an outbreak of TB in the north of Canada, the strain circulating during the outbreak year was detected even in people with one or more previous episodes of active TB disease in the preceding two decades.^32^ As TB incidence declines, a greater proportion of disease is due to a remote infection.^1^ ^5^

Recognising that the incubation period of TB is generally shorter than previously thought has important implications for vaccine trials. It allows for short vaccine efficacy trials, as has already been demonstrated for BCG and the vole vaccine, which showed early benefits in the MRC trial conducted in England and Wales (1951-70) (supplementary figure 4).^21^

As clinicians caring for patients in low transmission countries, we predominantly see TB disease in elderly people who were infected at a time when TB was widespread (remote infection) and in immigrants from high transmission areas and their contacts (recent or remote infection).^6^ ^7^ ^33^ In such low burden areas, screening contacts of patients with TB and others known or likely to be recently infected is important to prevent outbreaks. Biomarkers that distinguish newly acquired infection from remote infection could help to prioritise interventions.^34^ ^35^ But in high transmission countries, preventive therapy for contacts is of limited value given the high continuous chance of re-infection from known and unknown contacts. The latest WHO guidelines recognise this.^36^

Although the biology of latency is tantalising, the importance of this phenomenon for global TB control and for prioritising research is less convincing. That approximately 10 million new cases of TB disease are diagnosed each year is well cited, but we cannot find a published estimate of the number of people who are newly infected each year, even though these people are at the highest risk of developing disease. Based on the number of contagious cases worldwide, we can confidently assume that there are tens of millions of new infections per year, if not more.

WHO has pledged to eliminate TB by 2035 through its End TB Strategy.^37^ Its staged implementation plan includes “new tools: a vaccine, new drugs, and treatment regimens for treatment of active TB disease and latent TB infection,” which again reflects the concern that the large reservoir of people with latent TB infection may stymie efforts to achieve this goal. We hope that the evidence that most TB cases occur within 18-24 months of infection will lead to reconsideration of the current strategy. If focused attention was given to those with active TB disease and their newly infected contacts, TB elimination might be achieved sooner than projected.

**Key messages**

The current thought is that *Mycobacterium tuberculosis* frequently establishes a latent infection following which there is a reactivation process that leads to active TB disease, after a long and variable incubation period
Rather, the incubation period of TB is typically several months to two years, and after that, disease is relatively infrequent
There is no evidence for a bimodal distribution of TB that distinguishes primary progressive TB from reactivation TB
Immunoreactivity to TB does not necessarily indicate the presence of live bacteria, as reactivity can persist after infection has been cleared
Classifying two billion people with evidence of immunoreactivity as having latent TB infection may divert fundamental research and public health interventions away from transmissible active TB disease and newly infected people at highest risk of progression to disease

## Web extra

Extra material supplied by authors

**Appendix:** Supplementary boxes and figures

## Notes

Contributors and sources: The contributors are clinician-scientists with a longstanding interest in the pathogenesis and epidemiology of TB. MAB is an expert in clinical microbiology, infectious diseases, bacterial genomics and molecular epidemiology of TB. PHE is an expert in clinical microbiology and infectious diseases. LR is an expert in infectious diseases and the immunopathogenesis of mycobacterial infections. All authors searched the literature, wrote, and edited the paper and designed the figures; PHE prepared the figures. The sources of information used include a contemporary literature search using PubMed and Google Scholar, a search of older articles from the pre-antibiotic era and a request to many colleagues in the field for counterfactual reports that challenge the content and/or message of this manuscript. LR is the guarantor.

Funding: This work was funded by a Foundation Grant from the Canadian Institutes for Health Research (MAB) and the Wellcome Trust and National Institute of Health Research Cambridge Biomedical Research Centre and the National Institutes of Health (LR). The authors’ funders had no influence on this article.
