# Supplementary material for: Revisiting the timetable of tuberculosis
Source: BMJ. 2018 Aug 23;362:k2738. doi: 10.1136/bmj.k2738 (PMC6105930; doi:10.1136/bmj.k2738)

**Supplementary box 1—Definitions/Statements From Authoritative Bodies and Online Medical Resources**

**World Health Organization (WHO)**

**http://www.who.int/tb/challenges/ltbi_faqs/en/**

Latent TB infection (LTBI) is a condition in which TB bacteria (*M. tuberculosis*) survive in the body in a dormant state.

http://www.who.int/tb/publications/2018/latent-tuberculosis-infection/en/

Latent tuberculosis infection (LTBI) is "defined as a state of persistent immune response to prior-acquired *Mycobacterium tuberculosis* antigens without evidence of clinically manifested active TB, affects about one-third of the world's population. "

**Canadian TB Standards**

(http://www.phac-aspc.gc.ca/tbpc-latb/pubs/tb-canada-7/assets/pdf/tb-standards-tb-normes-appa-eng.pdf)

*Latent tuberculosis infection (LTBI)* The presence of latent or dormant infection with *Mycobacterium tuberculosis*. Patients with LTBI have no evidence of clinically active disease, meaning that they have no symptoms, no evidence of radiographic changes that suggest active disease and negative microbiologic tests; they are non-infectious.

*Primary tuberculosis* This includes primary respiratory tuberculosis and tuberculous pleurisy in primary progressive tuberculosis.

*Reactivation tuberculosis* The development of active disease after a period of latent tuberculosis infection. In Canada, the term "reactivation" tuberculosis was previously used to refer to a recurrence.

*Post-primary tuberculosis* older term – see reactivation tuberculosis.

**Centers for Disease Control and Prevention (CDC)**
(https://www.cdc.gov/tb/publications/faqs/pdfs/qa.pdf)

*Latent TB infection* In most people who breathe in TB bacteria and become infected, the body is able to fight the bacteria to stop them from growing. The bacteria become inactive, but they remain alive in the body and can become active later . This is called latent TB infection .

*TB disease* If the immune system can't stop TB bacteria from growing, the bacteria begin to multiply in the body and cause TB disease.

**National Institutes of Health Statement on World TB Day, March 24, 2018**

(https://www.niaid.nih.gov/news-events/nih-statement-world-tuberculosis-day-march-24-2018)

According to the WHO, more than 2 billion people globally are "latently" infected with TB, meaning they carry the bacteria but are currently without symptoms.

Up to 13 million people in the United States are estimated to have latent TB infection, according to the US Centers for Disease Control and Prevention.

**National Institute for Health and Care Excellence (NICE)**

(https://www.nice.org.uk/guidance/ng33/resources/tuberculosis-pdf-1837390683589)

*Latent TB* The initial infection clears in over 80% of people but, in a few cases, a defensive barrier is built round the infection and the TB bacteria lie dormant. This is called latent TB; the person is not ill and is not infectious.

**Supplementary box 2—Funding for TB latency**

2001 NIH RFA

Title "Response to the Presidential Vaccine Initiative - Overcoming the Tuberculosis Latency Challenge" (https://grants.nih.gov/grants/guide/rfa-files/RFA-AI-01-009.html)

Wording of call "The objective of this Request for Applications (RFA) is to stimulate investigator-initiated research to elucidate the mechanisms underlying persistent, asymptomatic infection (also referred to as "latency") with *Mycobacterium tuberculosis*. … A better understanding of persistent infection is an essential step toward developing improved intervention strategies to eliminate tuberculosis as a public health problem."

2005 BMGF Grand Challenge in Global Health, a family of initiatives fostering innovation to solve key global health and development problems (https://gcgh.grandchallenges.org/)

One of the 43 funded grants was entitled "Drugs for treatment of latent tuberculosis" with the following abstract: An estimated 2 billion individuals—a third of the world's population—have been exposed to Mycobacterium tuberculosis (MTB) and carry the infection in its latent form, retaining a lifelong risk of developing TB disease. Programs to control tuberculosis now focus on childhood vaccination and treatment for people with active disease. Reversing TB's spread, however, requires an intervention that will prevent disease in those who are already infected. The lack of knowledge about the biology of latent TB infection stands in the way of the development of such an intervention. [An] international team of researchers from the U.K., U.S., Singapore, Korea, and Mexico is attempting to further elucidate the fundamental biology of latency and use this knowledge to develop drugs against latent TB.

2008 BMGF Grand Challenges Explorations (https://gcgh.grandchallenges.org/challenge/explore-basis-latency-tuberculosis-round-1 and https://gcgh.grandchallenges.org/challenge/explore-basis-latency-tuberculosis-round-2).

Call for LTBI proposals: " ROADBLOCK: Most humans exposed to *Mycobacterium tuberculosis* contain the infection in an asymptomatic latent form. One third of the world's population is estimated to have latent TB, representing a vast reservoir from which active disease and subsequent transmission propagates. Interventions that identify and eliminate latent infection might break the cycle of disease transmission and reverse the TB epidemic."

Of the 1313 awards made, 15 were to study TB latency (https://gcgh.grandchallenges.org/grants?f[0]=field_challenge%253Afield_initiative%3A37073&f[1]=field_challenge%253Afield_short_title%3ATuberculosis%20Latency)

**Supplementary figure 1**—Time to development of active tuberculosis in TST or IGRA positive contacts of patients with active tuberculosis. A. Total population of 739 people. B. Fraction of contacts developing tuberculosis over time by age group. The incubation time is the time since the tuberculosis diagnosis of the index case. Redrawn from data in Sloot, et. al, 2014.[20]

A

B

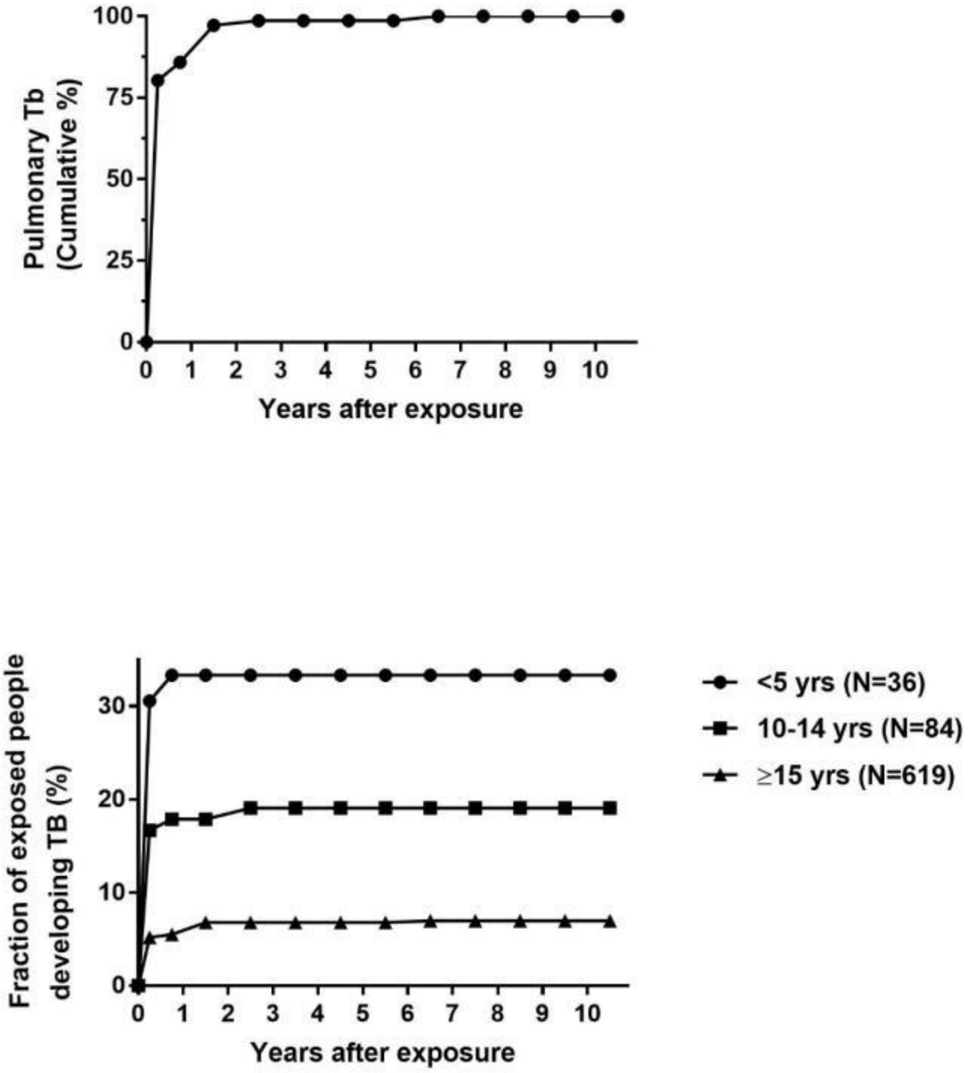

**Supplementary figure 2**—Incubation time to onset of active tuberculosis in a 48 person tuberculosis outbreak inferred by whole genome sequencing of isolates and mutation rates of isolates. Data redrawn from Hatherell, et. al, 2016.[19]

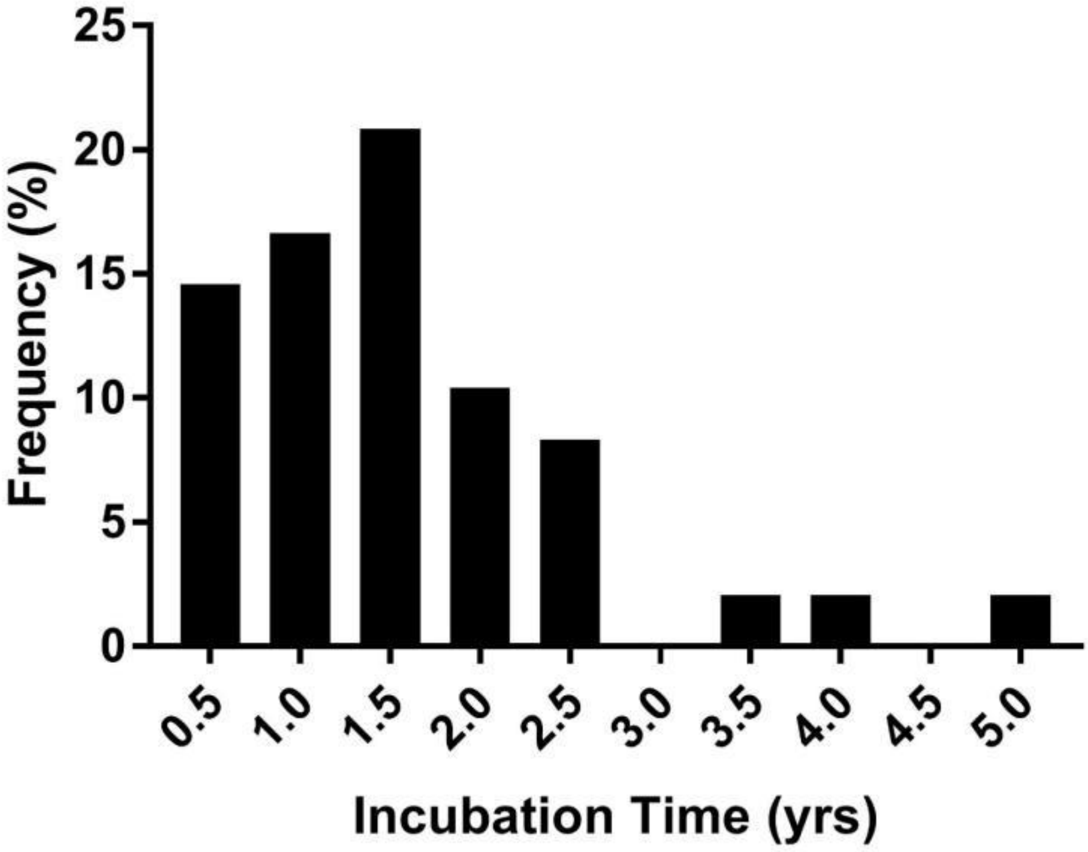

**Supplementary figure 3**—Rates of tuberculosis acquisition in nursing students after three years of exposure to patients with tuberculosis during their training period, by pre-exposure tuberculin skin test result. Error bars show 95% confidence intervals. Data from Heimbeck [28]

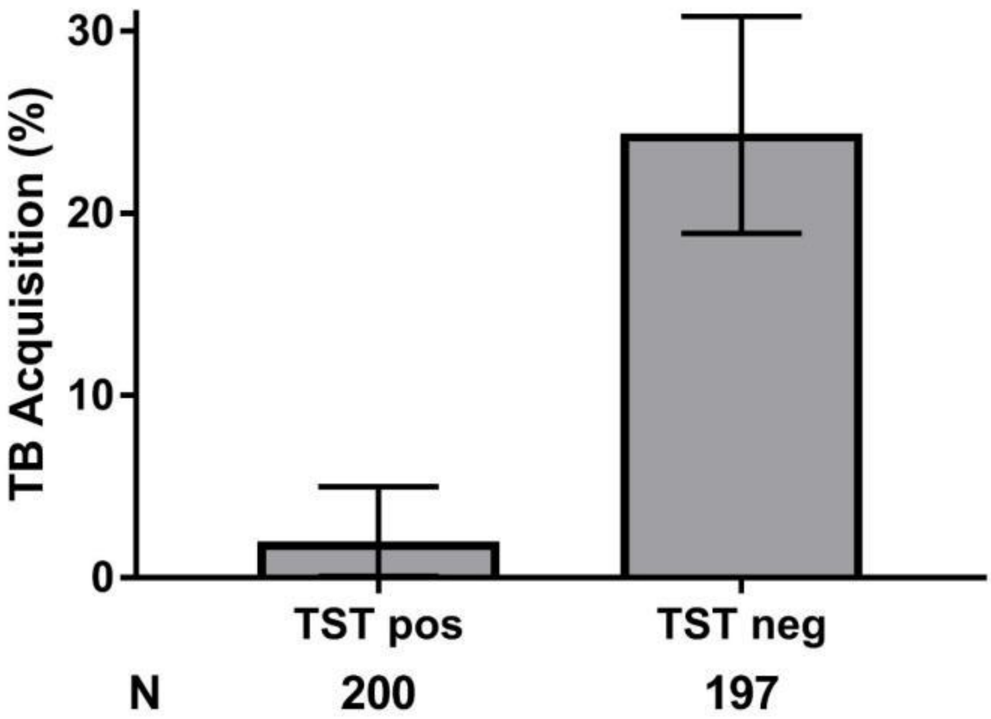

**Supplementary figure 4**—The incidence of active TB in 12 867 British adolescents who were TST negative, observed over 20 years, from 1951 to 1970, contrasted with the incidences of active TB in TST negative adolescents who received either BCG (n=13 598) or vole bacillus (*Mycobacterium microti*) (n=5,817) vaccines. Data from D'Arcy Hart and Sutherland.[21]

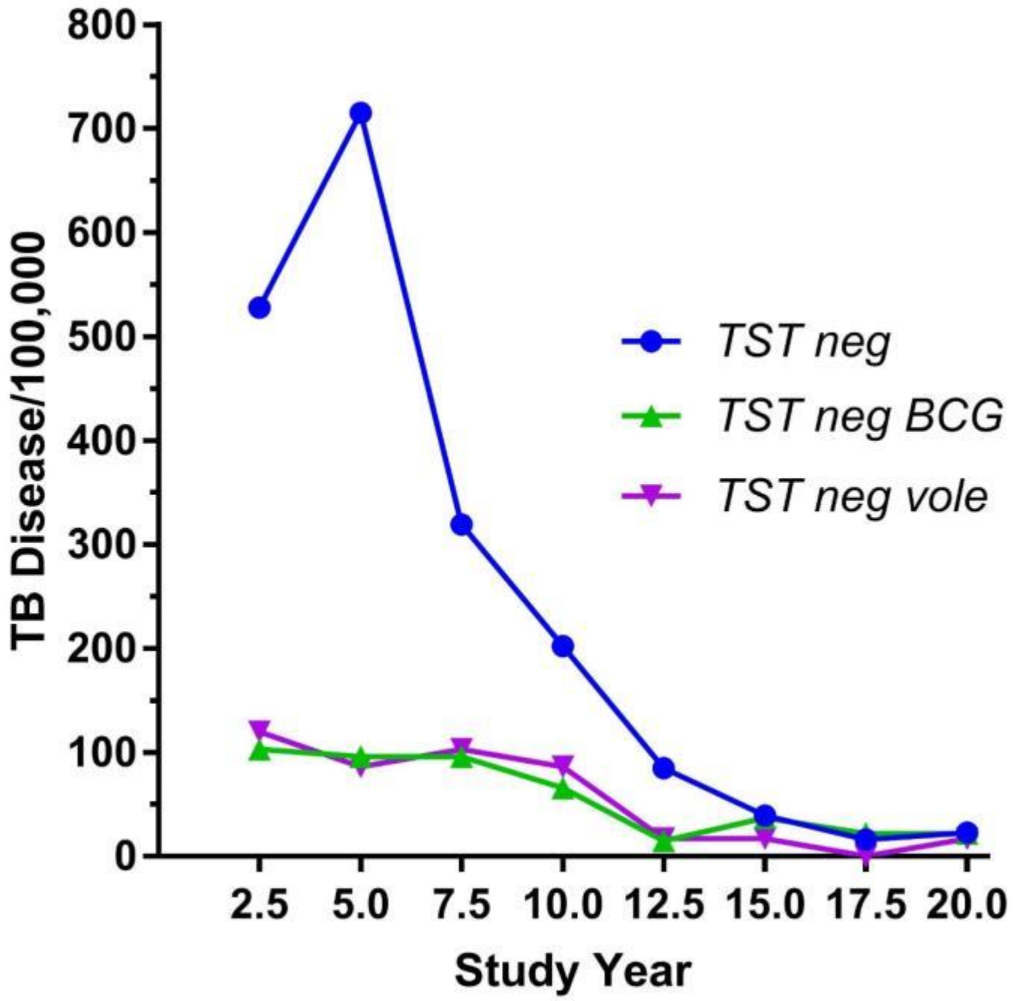

Supplement: Supplementary file 1 — Appendix: Supplementary boxes and figures [file raml045033.ww1.pdf]
